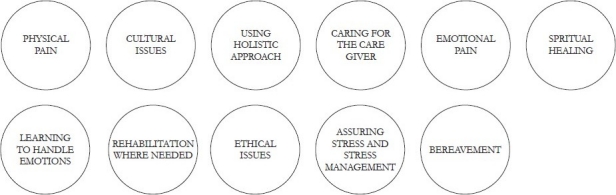# Total Pain Management

**DOI:** 10.4103/0973-1075.76246

**Published:** 2011-01

**Authors:** Agnes Panikulam

**Affiliations:** Holy Family Hospital Campus Okhla Road, Medical Mission, Sisters Joyti Bhawan New Delhi, India

**Keywords:** Total pain management, Team approach, Multidisciplinary team, Holistic care

## Abstract

We CanSupport provide holistic care to the patients and family. This means, physical, emotional, psychosocial and spiritual care. The objective of this article is to implement a plan for improved high quality care, within a dynamic and complex health care system for palliative care. Twelve years of working experience with palliative care in CanSupport ‘India’ and 10 years of working palliative care aboard (USA). High level satisfaction of the patient of the patient and families due to the psycho, socio, spiritual model and help for income generation and vocational training. We suggest and encourage, to we this model for all palliative care centre and institutions

We at CanSupport provide home based palliative care to cancer patients in the national capital region of Delhi. Our services are free and main focus is marginalized sections of our society. We have 7 field centers and 11 home care teams. Each home care team consists of a doctor, nurse and a counselor. Nurse is a backbone of the team and act as a team coordinator. The team members provide holistic care to the patients and family this means, physical, emotional, psychosocial and spiritual care to the patients and family. We firmly believe that for total pain management and quality care requires much disciplinary team approach using medico- psychosocial model.

Our care is patient focused and family oriented to take care of

- Physical
- Emotional
- Psychosocial
- Spiritual issues

through a Multidisciplinary Team chiefly consisting of

- Nurse
- Physician
- Counselor

and complemented, as required, by

- Volunteers
- Social workers
- Medical specialists
- Psychologists
- Psychiatrists
- Physiotherapist/Occupational therapist
- Recreational therapist (Complementary therapist)
- Nutritionist
- Spiritual/Clergy member (priest, chaplain)
- Social worker and vocational counselor, etc.

When a member of the family get sick, the whole family is affected. The family’s functional equilibrium is lost and the emotional shock vibrates through the entire system. Therefore, it is important to handle the patient and family by a multidisciplinary team. Medical group-doctor and nurse need to implement a plan for improved high quality care, within a dynamic and complex health care system. Members are committed to collaborate through a multidisciplinary team. They initiate a relationship with palliative care patients and family and coordinate the assessment and intervention for each patient.

Besides focusing on the patient by the medical team, counselors have a specific task to accomplish-study the history of the family stress level, grief patterns, communication, dynamic among the members, family support, belief system emotional expressed by the members and many other interactions within the family. After observation, counselor and patient along with the family, look at the range of problems present and prioritize them. This list is revised on a regular basis as, social, emotional, and spiritual issue changes can develop. The experiences of the total pain can add to individual’s fear, shame, anger and quilt. Therefore, counselors need to focus on the psychologically appropriate interventions to address the issues.

A multidisciplinary team facilitates team members to directly interact with patients and family and also shares information among the members. They work together to achieve the goal. An effective team requires collaboration, coordination and leadership and above all maturity in decision making.

When a person enters the final stage of the dying process, many major dynamics are at work-physical, emotional, and spiritual. On the physical side, body begins the final stages of shutting down; emotionally, patient might experience feelings of fear, anger, guilt, etc. The spirit of the person begins its final journey aimed at releasing the body from all attachment. The release follows its own priorities, which may include, setting unfinished business and seeking permission from the family to let go. This process needs to happen in a way appropriate to the values, beliefs, and life style of the dying person. Therefore, it is important to have a holistic approach.

Our past 13 years of experience shows that a multidisciplinary team approach using the Medico-psycho-socio-spiritual model is very effective.

## Benefits of the model

1. Total pain management by an expert team
2. Focus on the whole person- body mind and spirit
3. Pain relief in all level
4. Promoting quality care
5. Increase in support system
6. Opportunity to resolve unresolved issues
7. Psycho-socio, spiritual issues are handled
8. Understanding that if care is not possible healing is possible
9. Creating peace and harmony within on self and others
10. High level satisfaction

**Table:** 

Medical model	Medico-psycho-sociospiritual model
Focus on diseases only Most of the time, individual approach to evaluate Respond to physical pain Pain relief in physical level Support system low level Low level satisfaction	Focus on the whole person body, mind, and spirit Team approach to evaluate Respond to total pain Pain relief in all levels Increase in support system High level satisfaction

**Figure:**